# Reproductive lifespan in association with risk of hypertension among Chinese postmenopausal women: Results from a large representative nationwide population

**DOI:** 10.3389/fcvm.2022.898608

**Published:** 2022-08-08

**Authors:** Zhen Hu, Lu Chen, Xin Wang, Linfeng Zhang, Zuo Chen, Congyi Zheng, Xue Cao, Yuxin Song, Haoqi Zhou, Yixin Tian, Jiayin Cai, Yilin Huang, Runqing Gu, Ye Tian, Lan Shao, Zengwu Wang

**Affiliations:** ^1^State Key Laboratory of Cardiovascular Disease, Division of Prevention and Community Health, National Center for Cardiovascular Disease, National Clinical Research Center of Cardiovascular Disease, Fuwai Hospital, Peking Union Medical College and Chinese Academy of Medical Sciences, Beijing, China; ^2^School of Population Medicine and Public Health, Chinese Academy of Medical Sciences and Peking Union Medical College, Beijing, China

**Keywords:** CHS, Chinese Hypertension Survey, postmenopausal women, reproductive lifespan, hypertension, risk, blood pressure

## Abstract

**Background:**

The association between reproductive lifespan and risk of hypertension among postmenopausal women is unclear.

**Methods:**

A total of 94,141 postmenopausal women with a mean age of 64.8 years from the China Hypertension Survey were enrolled at baseline from 2012 to 2015. A standardized questionnaire was used to collect relevant information by well-trained interviewers. Blood pressure and physical examination of the participants were performed by trained medical staff. Logistic regression was used to estimate the odds ratios for hypertension by years of reproductive lifespan.

**Results:**

The average years of reproductive lifespan in Chinese women was 34.0 years. Women who were longer in reproductive lifespan were more likely to have older age at recruitment, higher body mass index, larger waist circumference, lower mean systolic blood pressure, and higher mean diastolic blood pressure (*p* < 0.05). After adjustments, odds ratios (95% confidence interval) for hypertension were 1.035 (0.988–1.085), 1.007 (0.966–1.048), 1.000 (reference), 0.932 (0.899–0.967), and 0.953 (0.909–0.997) for those with reproductive lifespan at ≤ 28, 29–31, 32–34 (reference), 35–37, and ≥ 38 years, respectively, with a significantly inverse association was seen in those with reproductive lifespan at 35–37 and ≥ 38 years. The overall risk of hypertension declined with the increase in reproductive lifespan, and the risk of hypertension was reduced by 1.1% for every 1-year increase in the reproductive lifespan (odds ratio, 0.989; 95% confidence interval, 0985–0.994) per year. The negative association between reproductive lifespan and hypertension was evident among age at recruitment groups, body mass index categories, and education levels, with the strongest association among women aged ≥ 70 years. Positive associations between reproductive lifespan and risk of hypertension were evident among women aged < 60 years, and this association was stronger among current alcohol drinkers.

**Conclusion:**

Based on the large nationally representative sample, Chinese postmenopausal women with a shorter reproductive lifespan have a higher risk of hypertension.

## Introduction

Hypertension is one of the most important and preventable risk factors for cardiovascular diseases, which has been recognized as a public health challenge worldwide (1–3). It is estimated that approximately 1.56 billion adults worldwide will be diagnosed with hypertension by 2025 (4). In China, the prevalence of hypertension is increasing from 13.6% in 1991 to 23.2% in 2012 (5, 6). Data from the China National Hypertension Survey Epidemiology Follow-up Study show a strong, linear, and significant association between SBP and DBP and risk for cardiovascular disease (CVD), coronary heart disease (CHD), and stroke, independent of other risk factors (7). Previous studies suggested that premenopausal women typically have lower blood pressure (BP) than men. However, the prevalence of hypertension is higher in postmenopausal women than that in men (8). Therefore, it is important to identify potential risk factors for the prevention of hypertension, particularly among postmenopausal women.

The age of menarche and menopause as the two endpoints of reproductive lifespan may have an implication for trajectory of hypertension later in life. However, the available evidence has remained unclear whether reproductive lifespan is an important risk factor for hypertension due to limited studies. Earlier studies showed inconsistent results on the effect of timing of menarche (9–12) and menopause (13–15) on hypertension risk with very few studies conducted overall. If such a link exists, then the duration of the reproductive lifespan from menarche to menopause might serve as an improved marker of hypertension risk in women. Given that hypertension is an important risk factor for CVD, clarifying the association between reproductive lifespan and the risk of hypertension in postmenopausal women could help identify subgroups of women at risk for hypertension and confirm those who should be targeted for early intervention, which is critical for preventing hypertension.

There is nothing of nationally representative large-sample studies to explore the relationship between reproductive lifespan and hypertension in Chinese women. The present study aimed to investigate the associations between reproductive lifespan and the risk of hypertension among postmenopausal women participating in the Chinese Hypertension Survey (CHS), a large, nationally representative sample study conducted in China.

## Materials and methods

### Study design and participants

**FIGURE 1:**
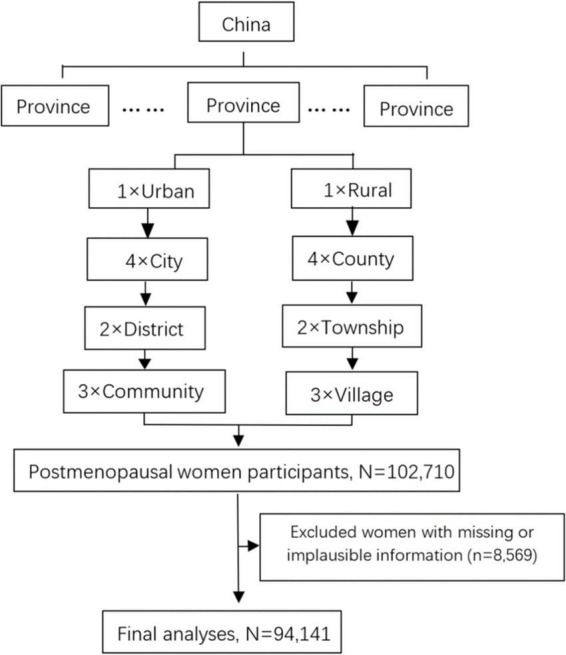
Chart of the subject inclusion and exclusion. PPS, probability proportional to size; SRS, simple random sampling.

All participants in this study were derived from the CHS, a nationally representative sample survey of the Chinese general population aged ≥ 15 years from all 31 provinces in the mainland conducted from October 2012 to December 2015. More details on CHS can be found in the previous publication (16). A stratified multistage random sampling method was used in the CHS. The first stage of sampling was to select four cities in urban areas and four counties in rural areas by using the probability proportional to size method within each province. Then, a simple random sampling method was used to select two districts or two townships in each city or county, and three communities or villages in each district or township. At the final stage of sampling, a certain number of participants from 14 sex/age groups (men and women aged 15–24, 25–34, 35–44, 45–54, 55–64, 65–74, and ≥ 75 years) were selected from communities using lists compiled by the local government registries of households. A total of 487,353 participants from 262 urban resident cities and rural counties were enrolled, of which 253,531 (52.02%) were women. Of the total sample of 102,710 postmenopausal women, 94,141 participants were included in the analysis after exclusions of 8,569 women with missing or implausible information (Figure 1).

Prior to recruitment, written informed consent was obtained from each participant. This study was approved by the Ethics Committee of Fuwai Hospital (Beijing, China).

### Measurement and data collection

Blood pressure on the right upper arm was measured three times by trained medical staff after the subjects rested for at least 5 min, with an interval of at least 30 s between each measurement with an observer present, using an OMRON HBP-1300 Professional Portable Blood Pressure Monitor (OMRON, Kyoto, Japan). The average of the three measurements was used for the analysis.

All subjects were interviewed by well-trained interviewers using standardized questionnaires developed by Fuwai Hospital, which covered social-demographic characteristics, lifestyle factors, disease history, and reproductive factors. The physical examinations were conducted by trained physicians and nurses. Height was measured without shoes using a standard right-angle device (accurate to 0.1 cm). Waist circumference was measured using a fixed measuring tape and at the midpoint of the line between the crest of the ilium and the inferior margin of the 12th rib with participants wearing thin clothing (to the nearest 0.1 cm). Body weight without wearing heavy clothing was measured using an OMRON body fat and weight measurement device (V-body HBF-371, OMRON, Japan). Body mass index (BMI) was calculated as weight/height^2^ (kg/m^2^).

### Definitions

The reproductive lifespan was defined as the interval between age at menarche and menopause. At baseline, each female subject was asked her age at the time of her first period, which was recorded as age at menarche. The menopausal status was defined as menstruation stopped for at least 12 months and no history of hysterectomy or oophorectomy, or current pregnancy or lactation, and the age at which menopause occurred was recorded at baseline. According to the Chinese guidelines for hypertension management, hypertension was defined as systolic blood pressure (SBP) ≥ 140 mmHg, and/or diastolic blood pressure (DBP) ≥ 90 mmHg, and/or use of antihypertensive medication within 2 weeks.

Educational levels were divided into three groups: elementary or below, junior high school, and high school or above. Criteria for overweight and obesity were considered as BMI ranging from 24.0 to 27.9 kg/m^2^ and BMI ≥ 28.0 kg/m^2^, respectively. Current smoking was defined as someone who has smoked at least 20 packets of cigarettes in their lifetime and still smokes. Current alcohol drinking was defined as consuming alcoholic beverages at least once per week in the past month. A family history of hypertension refers to one in which parents, siblings, or children have hypertension.

### Statistical analysis

Baseline characteristics of participants were presented as mean and standard deviation (SD) for normally distributed data or as a proportion for categorical data. Categorical variables were analyzed using the chi-square test, and continuous variables were analyzed by one-way analysis of variance (ANOVA). Logistic regression was used to estimate odds ratios (ORs) and 95% confidence intervals (CIs) for hypertension associated with reproductive lifespan (classified as ≤ 28, 29–31, 32–34, 35–37, and ≥ 38 years) with 32–34 years as the reference group. This study further investigated these associations by making additional adjustments for age at recruitment (continuous), ethnicity (Han or other ethnicities), region (urban or rural), education level (elementary or below, junior high school, high school or above), BMI (continuous), waist circumference (continuous), current smoking (yes or no), current alcohol drinking (yes or no), previously pregnant (yes or no), contraceptive use (yes or no), breastfeeding experience (continuous), stroke (yes or no), myocardial infarction (yes or no), and family history of hypertension (yes or no). We also examined the risk of hypertension in subgroups of women previously defined by age at recruitment, education level, and BMI. The threshold of statistical significance was set as *p* < 0.05. We used R 3.6.2 software to conduct our analyses.

## Results

The characteristics of the study participants are listed in Table 1. The mean (SD) age of study participants at recruitment was 64.8 (10.3) years, and the mean age at menarche, age at menopause, and reproductive lifespan were 15.7 (2.1), 48.9 (3.7), and 34.0 (3.3) years, respectively. The mean (SD) BMI, SBP, and DBP were 24.4 (3.8) kg/m^2^, 137.1 (21.5) mmHg, and 76.7 (11.0) mmHg, respectively. The proportions of women with reproductive lifespan at ≤ 28, 29–31, 32–34, 35–37, and ≥ 38 years were 12.3, 18.5, 29.3, 27.0, and 12.8%, respectively. A minority of women had an education level at high school or above (9.3%) and were current smokers (3.1%), current alcohol drinkers (5.7%), ever pregnant (3.1%), and users of contraceptives (5.2%). Women who were longer in reproductive lifespan were more likely to have older age at recruitment, higher BMI, larger waist circumference, and lower mean SBP and higher mean DBP (*p* < 0.05). Most women had been pregnant (96.9%) and had breastfed (95.4%).

**TABLE 1:** Characteristics of study participants by reproductive lifespan.

Characteristics	Reproductive lifespan (year)						
	≤28	29–31	32–34	35–37	≥38	*N* (Mean = 34.0)	*P*-value
Number (%)	11,567 (12.3)	17,418 (18.5)	27,639 (29.3)	25,446 (27.0)	12,071 (12.8)	94,141 (100.0)	0.624
Age at recruitment, mean (SD)	64.5 (10.9)	64.4 (10.6)	64.7 (10.5)	64.8 (10.0)	65.4 (9.3)	64.8 (10.3)	0.037
Han ethnicity, *n* (%)	9,802 (84.7)	15,042 (86.3)	24,178 (87.5)	22,547 (88.6)	10,590 (87.7)	82,159 (87.3)	0.624
Urban resident, *n* (%)	4,454 (38.5)	7,336 (42.1)	12,939 (46.8)	13,158 (51.7)	6,495 (53.8)	44,382 (47.1)	0.505
**Education level, *n* (%)**							
Elementary or below	5,543 (47.9)	7,464 (42.8)	11,261 (40.7)	9,255 (36.4)	4,074 (33.7)	37,596 (39.9)	0.873
Junior high school	5,405 (46.7)	8,806 (50.5)	13,992 (50.6)	13,261 (52.1)	6,324 (52.4)	47,788 (50.8)	0.624
High school or above	619 (5.3)	1,148 (6.6)	2,386 (8.6)	2,390 (9.4)	1,673 (13.8)	8,756 (9.3)	0.188
**Body measurements and lifestyle**							
Body mass index (kg/m^2^), mean (SD)	24.0 (3.8)	24.2 (3.8)	24.3 (3.8)	24.5 (3.8)	24.8 (3.8)	24.4 (3.8)	0.000
Waist circumference (cm), mean (SD)	82.2 (10.4)	82.8 (10.5)	83.2 (10.4)	83.6 (10.2)	84.1 (10.2)	83.2 (10.3)	0.000
Current alcohol drinking, *n* (%)	823 (7.1)	1,066 (6.1)	1,544 (5.6)	1,299 (5.1)	678 (5.6)	5,410 (5.7)	0.873
Current smoking, *n* (%)	385 (3.3)	622 (3.6)	822 (3.0)	734 (2.9)	349 (2.9)	2,912 (3.1)	0.873
**Reproductive characteristics**							
Ever pregnant, *n* (%)	11,339 (96.7)	17,114 (96.4)	27,143 (97.0)	24,942 (97.1)	11,831 (97.1)	92,369 (96.9)	0.624
Ever use of contraceptives, *n* (%)	547 (4.7)	901 (5.2)	1,418 (5.1)	1,323 (5.2)	756 (6.3)	4,945 (5.2)	0.624
Breastfeeding experience, *n* (%)	11,037 (95.4)	16,511 (94.8)	26,438 (95.6)	24,283 (95.4)	11,544 (95.6)	89,813 (95.4)	0.624
Age at menarche, mean (SD)	17.1 (2.3)	16.6 (2.02)	15.9 (1.7)	14.8 (1.6)	14.3 (1.7)	15.7 (2.1)	0.000
Age at menopause, mean (SD)	42.7 (2.8)	46.8 (2.1)	49.0 (1.7)	50.7 (1.6)	53.9 (2.4)	48.9 (3.7)	0.000
**Blood pressure**							
Hypertension, *n* (%)	5,822 (50.3)	8,735 (50.1)	13,953 (50.5)	12,591 (49.5)	6,211 (51.4)	47,312 (50.2)	0.624
SBP (mmHg), mean (SD)	137.7 (22.2)	137.5 (22.2)	137.4 (21.8)	136.3 (20.9)	136.8 (20.3)	137.1 (21.5)	0.037
DBP (mmHg), mean (SD)	75.8 (11.3)	76.2 (11.3)	76.8 (11.0)	77.1 (10.8)	77.5 (10.8)	76.7 (11.0)	0.000
Family history of hypertension, *n* (%)	3,272 (28.3)	5,190 (29.8)	8,230 (29.8)	7,740 (30.4)	3,860 (32.0)	28,292 (30.1)	0.624
**Other diseases**							
Stroke, *n* (%)	255 (2.2)	372 (2.1)	594 (2.1)	545 (2.1)	281 (2.3)	2,047 (2.2)	0.624
Myocardial infarction, *n* (%)	55 (0.5)	103 (0.6)	151 (0.6)	143 (0.6)	60 (0.5)	512 (0.5)	0.624

Percentages were calculated based on women with complete information for that specific variable; SD, standard deviation; BMI, body mass index; SBP, systolic blood pressure; DBP, diastolic blood pressure.

**FIGURE 2:**
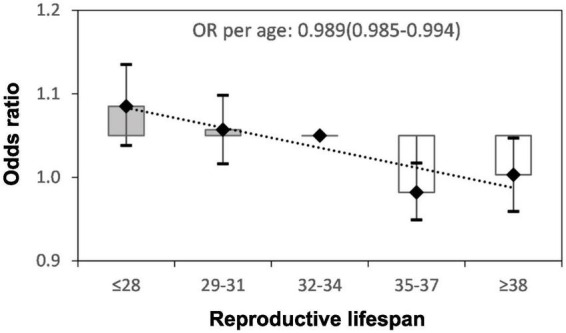
Adjusted odds ratio (OR) and 95% confidence interval (CI) for hypertension by the years of reproductive lifespan. Adjusted for age at recruitment, body mass index, waist circumference, region, ethnicity, education level, smoking, alcohol drinking, family history of hypertension, stroke, myocardial infarction, pregnant, contraceptive use status, and breastfeeding experience.

**FIGURE 3:**
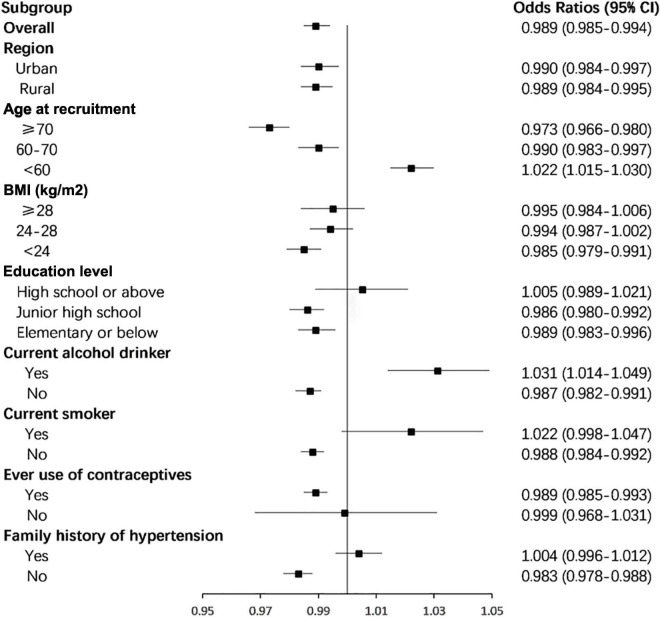
Subgroup analyses of the associations between total reproductive years and risk of hypertension according to possible influencing factors. Analyses were adjusted for age at recruitment, body mass index, waist circumference, region, ethnicity, education level, smoking, alcohol drinking, family history of hypertension, stroke, myocardial infarction, pregnant, contraceptive use status, and breastfeeding experience.

Figure 2 shows the adjusted ORs for hypertension by reproductive lifespan (≤28, 29–31, 32–34, 35–37, and ≥ 38 years). After adjustment for age at recruitment, ethnicity, region, education level, BMI, waist circumference, current smoking, current alcohol drinking, family history of hypertension, stroke, myocardial infarction, and reproductive factors, the ORs (95%CIs) were 1.035 (0.988–1.085), 1.007 (0.966–1.048), 1.000 (reference), 0.932 (0.899–0.967) and 0.953 (0.909–0.997) for those with reproductive lifespan at ≤ 28, 29–31, 32–34 (reference), 35–37, and ≥ 38 years, respectively. The significantly inverse association was seen in those with reproductive lifespan at 35–37 and ≥ 38 years. The overall risk of hypertension declined with the increase of reproductive lifespan. After adjustments, ORs (95%CI) of hypertension in subgroups of women defined by age at recruitment, BMI, and education level are shown in Table 2. Subgroup analyses were also performed based on age at recruitment, region, education level, smoking status, alcohol consumption status, BMI, menopausal status, use of contraceptives, and family history of hypertension (Figure 3). Reproductive lifespan was inversely associated with hypertension, with an adjusted OR of 0.989 (95%CI: 0985–0.994) per year. The risk of hypertension was reduced by 1.1% for every 1-year increase in reproductive lifespan. Negative associations between reproductive lifespan and risk of hypertension were evident among urban and rural women; women aged ≥ 70 and 60–70 years; women with BMI < 24kgm^2^; women with education level in junior high school and elementary or below groups; no current alcohol drinker, no current smoker, ever use of contraceptives, and no family history of hypertension. This association was strongest in women aged ≥ 70. Positive associations between reproductive lifespan and risk of hypertension were evident among women aged < 60 years and strongest among current alcohol drinkers.

**TABLE 2:** Association between reproductive lifespan and risk of hypertension.

Subgroups	Reproductive lifespan (year)						
	≤28	29–31	32–34	35–37	≥38	*N*	*P*-value
**By age at recruitment**							
< 60	0.955 (0.877–1.040)	0.992 (0.923–1.067)	Reference	0.985 (0.924–1.050)	1.058 (0.973–1.150)	1.022 (1.015–1.030)	<0.05
60–70	1.053 (0.973–1.140)	1.036 (0.967–1.110)	Reference	0.954 (0.897–1.016)	1.061 (0.984–1.154)	0.990 (0.983–0.997)	<0.05
≥ 70	1.055 (0.972–1.145)	0.971 (0.903–1.043)	Reference	0.856 (0.803–0.913)	0.732 (0.675–0.794)	0.973 (0.966–0.980)	<0.05
**By body mass index (BMI), kg/m^2^**							
<24	1.047 (0.982–1.117)	0.999 (0.944–1.058)	Reference	0.918 (0.871–0.968)	0.885 (0.827–0.948)	0.985 (0.979–0.991)	0.106
24–28	1.043 (0.962–1.130)	1.014 (0.946–1.087)	Reference	0.943 (0.887–1.002)	1.031 (0.957–1.111)	0.994 (0.987–1.002)	0.098
≥28	0.956 (0.843–1.085)	1.007 (0.905–1.121)	Reference	0.939 (0.856–1.030)	0.952 (0.851–1.065)	0.995 (0.984–1.006)	<0.05
**By education level**							
Elementary or below	1.009 (0.943–1.081)	1.013 (0.952–1.078)	Reference	0.937 (0.884–0.994)	0.905 (0.839–0.977)	0.989 (0.983–0.996)	0.206
Junior high school	1.052 (0.983–1.126)	0.977 (0.923–1.035)	Reference	0.913 (0.867–0.960)	0.928 (0.871–0.989)	0.986 (0.980–0.992)	<0.05
High school or above	1.006 (0.822–1.229)	1.138 (0.971–1.335)	Reference	1.028 (0.910–1.162)	1.200 (1.104–1.380)	1.005 (0.989–1.021)	<0.05

ORs were estimated using a logistic regression model and adjusted for age at recruitment, body mass index, waist circumference, region, ethnicity, education level, smoking, alcohol drinking, family history of hypertension, stroke, myocardial infarction, pregnant, contraceptive use status, and breastfeeding experience. Women with reproductive lifespan of 32–34 were used as the reference category.

## Discussion

Based on a nationally representative sample, we found that women with a reproductive lifespan of 35–37 and ≥ 38 years had decreased risk of hypertension. The longer the reproductive lifespan, the lower the risk of hypertension. The risk of hypertension was reduced by 1.1% for every 1-year increase in the reproductive lifespan. This association also appeared to be similar among subgroups. To our knowledge, this was the first comprehensive study to investigate the associations between reproductive lifespan and risk of hypertension in Chinese postmenopausal women. It is helpful to identify markers that are critical for preventing hypertension in women.

The interval between menarche and menopause defines a woman’s natural reproductive span. However, previous studies indicated both contradictory results about the effect of age at menarche and age at menopause on hypertension risk, and rare studies examined both factors during the reproductive lifespan. We found only one study conducted by Feng et al. that simultaneously examined the effects of age at menarche, reproductive lifespan, and menopause on the metabolic risk factors for cardiovascular diseases. The study found that every year’s increase in reproductive lifespan was associated with only a marginal increase in SBP and DBP and did not reach statistical significance (9). However, this study only selected a certain southern city in China as the population source, which cannot well represent the whole population of China. In addition, the study adjusted for only a small number of confounding factors, perhaps leading to a statistical bias.

Although several studies have explored the association between the risk of hypertension and age at menarche in women in China, these studies were limited to one province/city/area, included a smaller number of participants, and the conclusions of those studies were inconsistent (11, 17, 18). Therefore, using data from a representative sample of 234,867 Chinese women, our findings confirmed the association between age at menarche and the risk of hypertension, and showed that each 1-year delay in menarche was associated with a 6.2% increase in the prevalence of hypertension. The relationships between age at menarche and hypertension were also inconsistent in other countries. The result from 12,336 participants in the Korean National Health and Nutrition Examination Survey manifested no significant association between age at menarche and hypertension (19). However, a Brazilian study of 33,715 participants found that age at menarche was inversely associated with increased blood pressure (10).

There was limited and contradictory literature on the effect of age at menopause on the risk of hypertension among postmenopausal women. One study among middle-aged and older Chinese women showed that for each 1-year delay in menopause, the prevalence of hypertension decreased by 2% (20). Another study from China reported that the association between post-menopause and blood pressure disappeared after adjusting for BMI (9).

The mechanisms by which age at menarche or menopause is associated with hypertension are complex and are yet to be fully elucidated. The reproductive years can be regarded as the simplest measurement of cumulative exposure to sex hormones. A systematic review on the association between reproductive lifespan characteristics and risk of Type 2 diabetes and hypertension suggests a protective effect for a longer reproductive lifespan, while estrogen exposure may have an independent effect. In addition, various genes that regulate the renin-angiotensin system and adrenergic system, estradiol, testosterone, estrogen/androgen ratio, and environmental factors such as age, BMI, insulin, oxidative stress, and cholesterol affect the risk of hypertension among postmenopausal women (21). In particular, the decrease of estrogen level reduces the vascular relaxation effect of estrogen and improves the vasoconstriction effect of endothelin (22), leading to an increased risk of hypertension during the menopausal transition (23). Therefore, future longitudinal studies are needed to examine the relationship between reproductive markers and the risk of hypertension as well as the risk attributable to the administration of exogenous hormones, such as contraceptives and menopause hormone therapy (MHT).

Interestingly, we also found that the reproductive lifespan at 35–37 years was most associated with hypertension compared to > 38 years. In addition, when stratified by the age of recruitment, compared with 60–70 years and ≥ 70 years, positive associations between reproductive lifespan and risk of hypertension were evident among women aged < 60 years. Age is an important risk factor due to reproductive stages being part of the aging process. Considering both the timing of menarche and menopause during the reproductive lifespan, the study found that the effect of menopausal status disappeared after adjusting for age implying that much of the hypertension risk in menopausal transition was possibly related to biological aging (14). This explains the rational behavior of the phenomenon to some extent. In our study, to assess the effects of the reproductive lifespan on hypertension independent of the normal aging process, we removed the effect of age at recruitment before the analyses. This may result in underestimated effect sizes. Even in this case, we still get a significant effect that reflects a possible real association between reproductive lifespan and hypertension. We also discovered that the positive association between reproductive lifespan and hypertension was stronger among current alcohol drinkers in subgroup analyses. The alcohol-hypertension relationship has been well documented (24, 25). The study found that chronic exposure to alcohol alters the production of this same set of hormones (i.e., estrogen and testosterone), and hence, alcohol’s effects on the cardiovascular system could involve an indirect mechanism in which alcohol alters hormone levels and, in turn, the hormones affect the BP (26).

## Limitations and strengths

The important strength of our study is to explore the relationship between reproductive lifespan and hypertension in Chinese women for the first time in a nationally representative sample. In addition, standardized approaches and stringent quality control measures were also taken to ensure data quality and reliability. We adjusted for potential risk factors for hypertension and performed a detailed subgroup analysis to improve the reliability of the findings. Several limitations need to be considered. First, our participants were Chinese women, which minimized the confounding effects of ethnic background but might minish the generalization of our findings to other ethnic groups. Second, the reproductive lifespan was calculated based on age at menarche and menopause, which were self-reported and not based on medical documentation, raising the possibility of misclassification due to recall bias. However, previous studies have reported that the recall of age at menarche (27, 28) and menopause is relatively accurate (29, 30). Lastly, although we have fully adjusted for potential confounders, the possibility of residual confounding from other known or unknown risk factors cannot be completely ruled out. Finally, the cross-sectional design could not prove a causal relationship.

## Conclusion

Based on a representatively national sample of 94,141 Chinese postmenopausal women, we found that the longer the reproductive lifespan, the lower the risk of hypertension. The risk of hypertension is reduced by 1.1% for every 1-year increase in reproductive lifespan.

## Data availability statement

The original contributions presented in this study are included in the article/Supplementary material, further inquiries can be directed to the corresponding author.

## Ethics statement

The studies involving human participants were reviewed and approved by the Ethics Committee of Fuwai Hospital (Beijing, China). The patients/participants provided their written informed consent to participate in this study. Written informed consent was obtained from the individual(s) for the publication of any potentially identifiable images or data included in this article.

## Author contributions

ZH: conceptualization and writing – original draft preparation. LC: methodology, formal analysis, and software. XW: investigation and writing – original draft preparation partly. ZC, LZ, and CZ: data curation, formal analysis, and software; HZ, XC, YT, JC, YH, RG, YT, and LS: investigation. ZW: conceptualization, funding acquisition, and writing – review and editing. All authors contributed to the article and approved the submitted version.

## Abbreviations

BP: blood pressure
CHS: Chinese Hypertension Survey
CVD: Cardiovascular disease
SBP: systolic blood pressure
DBP: diastolic blood pressure
BMI: body mass index
SD: standard deviation
OR: odds ratio
CI: confidence interval
MHT: menopause hormone therapy.
