# Supplementary material for: Reproductive lifespan in association with risk of hypertension among Chinese postmenopausal women: Results from a large representative nationwide population
Source: Front Cardiovasc Med. 2022 Aug 8;9:898608. doi: 10.3389/fcvm.2022.898608 (PMC9393301; doi:10.3389/fcvm.2022.898608)
Supplement: Supplementary file 1 [file Table_1.docx]

| **Supplement Table 1. Risk for hypertension according to reproductive lifespan.** | | | | | |
| --- | --- | --- | --- | --- | --- |
| Model | Reproductive lifespan (year) | | | |  |
|  | ≤28 | 29-31 | 32-34 | 35-37 | ≥38 |
| Model 1 | 1.000 (0.956-1.045) | 0.999 (0.961-1.039) | 1.000 (reference) | 0.952 (0.919-0.986) | 1.008 (0.965-1.053) |
| Model 2 | 1.036 (0.988-1.085) | 1.008 (0.967-1.049) | 1.000 (reference) | 0.932 (0.899-0.967) | 0.953 (0.910-0.998) |
| Model 3 | 1.001 (0.957-1.046) | 0.998 (0.960-1.038) | 1.000 (reference) | 0.951 (0.918-0.985) | 1.004 (0.961-1.049) |
| Model 4 | 1.035 (0.988-1.085) | 1.007 (0.966-1.048) | 1.000 (reference) | 0.932 (0.899-0.967) | 0.953 (0.909-0.997) |

Model 1, adjusted for age at recruitment;

Model 2, adjusted for age at recruitment, body mass index, waist circumference, region, ethnicity, education level, smoking, alcohol drinking, family history of hypertension, stroke, myocardial infarction;

Model 3, adjusted for age at recruitment, pregnant, contraceptive use status, breastfeeding experience;

Model 4, adjusted for age at recruitment, body mass index, waist circumference, region, ethnicity, education level, smoking, alcohol drinking, family history of hypertension, stroke, myocardial infarction, pregnant, contraceptive use status, and breastfeeding experience.
